# Involvement of Pancreatic Stellate Cells in Regeneration of Remnant Pancreas after Partial Pancreatectomy

**DOI:** 10.1371/journal.pone.0165747

**Published:** 2016-12-09

**Authors:** Shigenori Ota, Miyuki Nishimura, Yuya Murakami, Naoko Kubo Birukawa, Akihiro Yoneda, Hiroki Nishita, Ryosuke Fujita, Yasushi Sato, Kenjiro Minomi, Keiko Kajiwara, Miyono Miyazaki, Maki Uchiumi, Shintaro Mikuni, Yasuaki Tamura, Toru Mizuguchi, Masafumi Imamura, Makoto Meguro, Yasutoshi Kimura, Koichi Hirata, Yoshiro Niitsu

**Affiliations:** 1 Department of Molecular Target Exploration, Sapporo Medical University, Sapporo, Japan; 2 Department of Surgery, Surgical Oncology & Science, Sapporo Medical University, Sapporo, Japan; 3 Department of Molecular Therapeutics, Center for Food & Medical Innovation Hokkaido University, Japan; 4 Hokkaido Laboratory of Molecular Therapeutics, Corporate Business Development Division, Nitto Denko Corporation, Hokkaido University, Japan; 5 Department of Medical Oncology and Hematology Sapporo Medical University, Sapporo, Japan; Centro Nacional de Investigaciones Oncologicas, SPAIN

## Abstract

**Background and objectives:**

Mechanism of regeneration of remnant pancreas after partial pancreatectomy (PX) is still unknown. In this study, effect of siRNA against the collagen specific chaperone, HSP47, which inhibits collagen secretion from activated pancreas stellate cells (aPSCs), and induces their apoptosis, on regeneration of remnant pancreas was determined.

**Methods:**

Pancreatectomy was performed according to established methods. Proliferation of cells was assessed by BrdU incorporation. Immunostaining of HSP47 was employed to identify PSCs. Progenitor cells were identified by SOX9 staining. Acinar cells were immunostained for amylase. Co-culture of acinar cells with aPSCs were carried out in a double chamber with a cell culture insert. siRNA HSP47 encapsulated in vitamin A-coupled liposome (VA-lip siRNA HSP47) was delivered to aPSCs by iv injection.

**Results:**

In remnant pancreas of 90% PX rat, new areas of foci were located separately from duodenal areas with normal pancreatic features. After PX, BrdU uptake of acinar cells and islet cells significantly increased, but was suppressed by treatment with VA-lip siRNA HSP47. BrdU uptake by acinar cells was augmented by co-culturing with aPSCs and the augmentation was nullified by siRNA HSP47. BrdU uptake by progenitor cells in foci area was slightly enhanced by the same treatment. New area which exhibited intermediate features between those of duodenal and area of foci, emerged after the treatment.

**Conclusion:**

aPSCs play a crucial role in regeneration of remnant pancreas, proliferation of acinar and islet cells after PX through the activity of secreted collagen. Characterization of new area emerged by siRNA HSP47 treatment as to its origin is a future task.

## Introduction

Evidence has accumulated indicating that the mature adult pancreas is capable of undergoing regeneration, similar to the liver, although to a lesser [1,2] extent.

In animals, mechanisms underlying this pancreatic regeneration imply; 1)neogenesis, which includes differentiation of progenitor cells to mature cellular components [3–11], or trans-differentiation of mature cells into other mature cell types; conversion of acinar cells toβ cells orαcell to βcell, and 2) self-replication of pre-existing exocrine [12] and /or endocrine [13,14] cells.

Such diversity in proposed mechanisms may be due to differences in experimental models or methodologies employed.

In a model such as one that total acinar and βcell loss was induced by diphtheria toxin[6,11], proof of self- replication of these cells was practically limited, and neogenesis could be the predominant mechanism. In less harsh models such as partial pancreatectomy [3,8,12,13] or pancreatic duct ligation [4,5,10,14], not only neogenesis from stem cell foci which are characterized by a number of tubule-like cells within massively deposited extracellular matrix (ECM) [3,8] but also self-replication of remnant mature cells could readily take place.

Further, in most of these animal experiments implications of the results with regard to the mechanism of regeneration could vary depending upon the markers chosen and timing of forced gene expression because lineage tracing methods utilizing cell type specific markers [5,12,14] were generally applied in these experiments to follow the conversion of cells.

Regardless of these discrepancies in mechanisms, numerous experiments conducted so far in animals, with no exception, assured plasticity and regeneration of both exocrine and endocrine cells in pancreas.

In humans, however, evidence for regeneration of endocrine components has been vague, albeit a small body of circumstantial evidence based on the observation of surgically resected pancreas suggested self replication of β-cells [15,16] or new β-cell formation [17]. In contrast, histological and functional recovery of exocrine components were definitively confirmed by long term observation of two consecutive pancreatectomy cases [16] and by histological analysis of double biopsied specimens, before and after the steroid therapy for autoimmune pancreatitis[18].

Nonetheless, in both animals and humans, investigations were mainly focused on the sources of cells which underwent regeneration, and little has been studied on the molecular mechanisms by which regeneration is triggered by extracellular stimuli although intracellular genetic events, expression of transcription factors which facilitate neogenesis, have been explored [19,20].

With regard to humoral factor(s) which may regulate regeneration of pancreas, most of the efforts were made to identify βcell proliferating substances [21] with no direct evidence of their activity during the recovery phase from tissue damage in vivo, and the molecular mechanisms of exocrine cell proliferation has been inadequately investigated.

Further inter-cellular networks which may regulate mature cell proliferation or neogenesis have not been adressed extensively.

Stellate cells were first identified as vitamin A storing star-shaped cells in the liver and subsequently were found to be distributed essentially to all organs, including pancreas [22].

When activated by inflammation with tissue damage, they proliferate and secrete collagen [22].

We have previously demonstrated that by employing vitamin A-coupled liposome, siRNA against collagen specific chaperone, (heat shock protein47; HSP47), the siRNA could be preferentially delivered to hepatic stellate cells or PSCs, thereby bringing about resolution of organ fibrosis in rat models [23,24].

We further revealed that those stellate cells treated with the siRNA-HSP47 underwent apoptosis, because they were dependent on self-collagen cleaved by membrane type1 matrix metalloproteinase (MT1-MMP) for their growth [25].

In the present investigation, utilizing siRNA HSP47 encapsulated VA-liposome, a potential role of PSCs in regeneration of rat pancreas after partially pancreatectomy is investigated

## Materials and Methods

### Rats

40 Six-week-old, male Sprague–Dawley (SD) rats and 6 Six-week-old, male SD-Tg (CAG-EGFP) rats with a weight range of 150g to 200g were used this experiment.

All rats were housed on a 12-hour light/dark cycle in Sapporo Medical University Animal Research Center. Animals were fed a laboratory diet with water and chow ad libtum. The rats were monitored once daily by a staff member in Sapporo Medical University Animal Research Center.

If one of the following conditions occurred during the experiment, animal was sacrificed and experiment termination considered; significant changes in physiological parameters such as breathing, heart rate, more than 15% weight loss and other signs of distress such as prolonged lying, aggressive behavior, anorexia.

We used somnopentyl as euthanasia measures based on the painful reduction guidelines on laboratory animal. Euthanasia treatment having been provided for 3 individuals.

All experiments followed the tenets and regulations of the Declaration of Helsinki, Cartagena protocol, and the animal experimental guidelines of Sapporo Medical University, and were performed with approvals from the Animal Care and the Institutional Ethics Committees of Sapporo Medical University.

### Partial pancreatectomy

Partial pancreatectomy on SD rat was performed under systemic anesthesia, using a Gemini Cautery kit and a swab as described earlier by Lehv et al [1], paying particular attention to avoid any damage to communicating vessels.

### Pancreatic stellate cells

Isolation of pancreatic stellate cells (PSCs) was performed, essentially based on the method by Apte et al. with minor modification to use 0.02% collagenase(Yakult co Lt Japan) for digestion of pancreas tissue, and 13.5% Nycodenz (Axis-Shield plc Scotland) for centrifugation.

The isolated PSCs were cultured 24hrs in DMEM 10%FCS to remove non-adhesive cellular components, then further cultured 5 days before use.

### Acinar cells

Pancreas tissue was treated with collagenase as described above in a PSC section and filtered through a 58–64 mm pore cell strainer to remove large clots of debris.

Then the filtrated cell coagulation was teared off by treatment with 0.25% trypsin/EDTA solution at 37°C for 15min, followed by filtration through a 100μm pore strainer. Thus obtained cell suspension was placed on density-gradient centrifugation with 13.5% Nycodenz and pellet cells were collected, removing PSCs in the middle layer.

After incubation in 75cm^2^-flask for 6hours in RPMI1640 supplemented with 10%FBS, 100nM Dexamethasone, (Sigma US), 0.1mg/ml soy bean trypsin inhibitor (Sigma US), 0.01mg/ml Aprotinin(TaKaRa Bio Inc Japan), 50um b-ME,5μg/ml, Insulin(Sigma US),the cells were added with 80% percoll to remove floating dead cells. The remaining cells were used as pancreas acinar cells (PACs).

### Preparation of VA-liposome siRNA HSP47 and VA-liposome siRNA HSP47 Cy5

VA-lip siRNA HSP47 and VA-lip siRNA HSP47 Cy5 (Kindly provided by Nitto Denko Techincal US) were prepared as described previously [23,24].

### Transfection of PSCs with siRNAHSP47

The PSCs were transfected with siRNA HSP47 with the same the method as applied for hepatic stellate cells [24,25].

### Quantitative reverse transcription polymerase chain reaction (qRT-PCR)

qRT-PCR for HSP47 in PSCs were carried out as described earlier [25].

### Administration of VA-lip siRNA HSP47 and VA-lip siRNA HSP Cy5

3mg/kg siRNA HSP47 or siRNA HSP47 Cy5 encapsulated in VA-lip was injected to SD rats through tail vein with the protocols described in S2 Fig.

### BrdU administration to SD rat

0.1mg/gBW BrdU (Sigma US), which was pre-diluted with PBS to 20mg/ml, was intraperitoneally administrated to SD rats, 3hours prior to harvesting pancreas tissue.

### FACS analysis of pancreas cells

Cells isolated from the pancreas were fixed in 4% paraformaldehyde for 30min at room temperature.

Cell permeabilization was performed either with 0.1% sodium citrate/0.1% Triton-X on ice for 1min for α-SMA staining of PSCs, or with 2 N HCl in PBS at room temperature for 5min for staining of amylase and BrdU of PACs. After blocking with 5% goat serum for 30min at room temperature, the cells were allowed to react with anti α-SMA rabbit antibody (Abcam US), anti-amylase rabbit antibody(Cells Signaling US) or anti-BrdU mouse antibody (MBL US) and subsequently secondary antibody, alexa 405 conjugated anti-rabbit IgG goat antibody (Invitrogen US) and alexa 647 conjugated anti-mouse IgG goat Antibody (Invitrogen US) for 30min on ice.

FACS analysis of these cell was carried out using FACS canto Ⅱ instrument with BD FACS diva software (Becton Dickinson US version 6.1.3).

### Immunohistological staining of pancreas tissue

Paraffin-embedded slices in citric acid were activated by autoclaving at 120°C 20min for staining of αSMA, BrdU and HSP47 and the slices in Histo VT one (Nacalai tesque Japan) were activated by heating at 90°C for 40 min, Incubation of the slices with primary antibodies against Nestin (Abcam US), SOX9 (Millipore Germany), amylase (Cells Signaling US) and HSP47 (Abcam US) carried out at 4°C overnight, and with secondary antibodies, HRP or AP conjugated anti-mouse or anti-rabbit IgG goat antibody (KPL US) at 37°C for 1h.

### Co-culture of PSCs and acinar cells

Details of procedure are shown in S4 Fig.

In brief, PACs and PSCs were isolated from normal and GFP rats respectively by the method described above.

Co-culture was carried out in double chamber with cell culture insert on which PSCs were plated and cultured for 24hrs, followed by an over lay of PACs to culture additional 12hrs.

BrdU was added 1hr prior to the termination of co-culture and BrdU uptake by non-GFP cells were measured by FACS.

### Quantification of fibrotic area and density of HSP47-positive Cells

Tissue specimen stained with Azan-Mallory method and immunostained for HSP47 were automatically photographed at four-fold magnification by Bz 9000 (Keyence corporation Japan), and jointing of each picture was also automatically carried out by BzⅡ analyzer software. Then we selected regions of blue stained collagen fibrils and the dark-brown DAB-stained HSP47-cells, to make enhanced contrast. Actual contrast enhancement was done by conversion of full color image into monochrome image(Binarization) utilizing BzⅡ analyzer software(Keyence corporation Japan).

Thus converted monochrome area was computed for quantification using BzⅡ analyzer software. Measurement was repeated three times based on color crossing to obtain the average number.

### Quantification of BrdU positivity

BrdU positive dots were visually counted per 8000 nuclei of one specimen for PACs, per all nuclei of each 2 specimen for islet cells and per 2000 nuclei of one specimen for ductule-like cells in one rat.

Such counting was carried out on specimen from 5 rats and average number of 5 rats was expressed as BrdU uptake rate percentage of BrdU positive PSCs was cultured as a ratio of number of BrdU positive PSCs per all PSCs in full field of two histological specimens.

### Statistical analysis

The unpaired *t*-test (two-sided) was used to compare the two groups, and the Tukey-Kramer method was used to compare multiple groups. Pearson’s correlation coefficient test was used to examine correlations between the two groups. The MedCalc software package (Ver 8.0.1.0; MedCalc Software bvba Belgium) and the SPSS program (Ver 22; SPSS Inc. US) wre used for calculations. The images of the tissue specimen stained by Azan-Mallory method and immunostained for HSP47 in foci area were obtained by BZ-X710 (Keyence corporation, Japan) with PlanApo_λ 20x NA = 0.75 (Nikon, Japan). The area of blue stained collagen fibrils and the dark-brown DAB-stained HSP47-cells were quantified using StrataQuest Analysis Software (TissueGnostics, Austria). The chi-squared test was used to compare the two groups for the percentage of BrdU positive PSCs. All data are shown as mean±standard deviation. A P value of<0.05 was considered statistically significant.

## Results

### Gross appearance and histological features of remnant pancreas after 90% and 70% resection

Although in previous reports, generation of foci which are considered to be the sites of progenitor cell proliferation after PX, has been described [3,8], the relative proportion of foci to duodenal area or its location in remnant pancreas were not clearly investigated.

**Fig 1:**
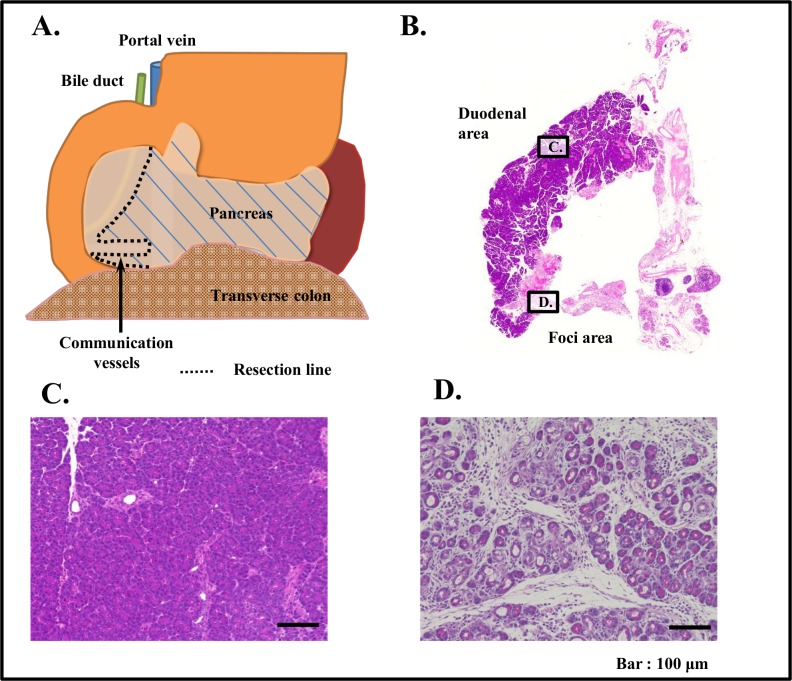
Schematic drawing of PX procedure, and gross appearance and histological feature of remnant pancreas. (A) Schematic drawing of remnant pancreas of 90% PX (rat). 5 rats were used for this experiment. Gross and histological appearances of these 5 rats were essentially the same. Thus representative gross appearance of remnant pancreas from 90% PX rat is shown in (B). (C)(D) Representative histological images of HE stained tissue specimen of duodenal area (C) and foci (D) of remnant pancreas from 90% PX rat. Note totally distinct histological feature of C from D.

On 5 rats that underwent 90% PX showed gross appearances and histological features of the remnant portion of the pancreas, showed essentially similar. Representative examples are shown in Fig 1.

The remnant pancreas of 90%PX rat (Fig 1A and 1B) in addition to an apparently normal pancreas areas at duodenal sites (Fig 1C), foci were clearly seen at the loci (Fig 1D) adjacent to communicating vessels, even though the proportion of foci to duodenal area was relatively small.

### Immunohistochemical analysis of amylase, insulin and SOX9 in duodenal and foci of remnant pancreas of 90% PX rat

**Fig 2:**
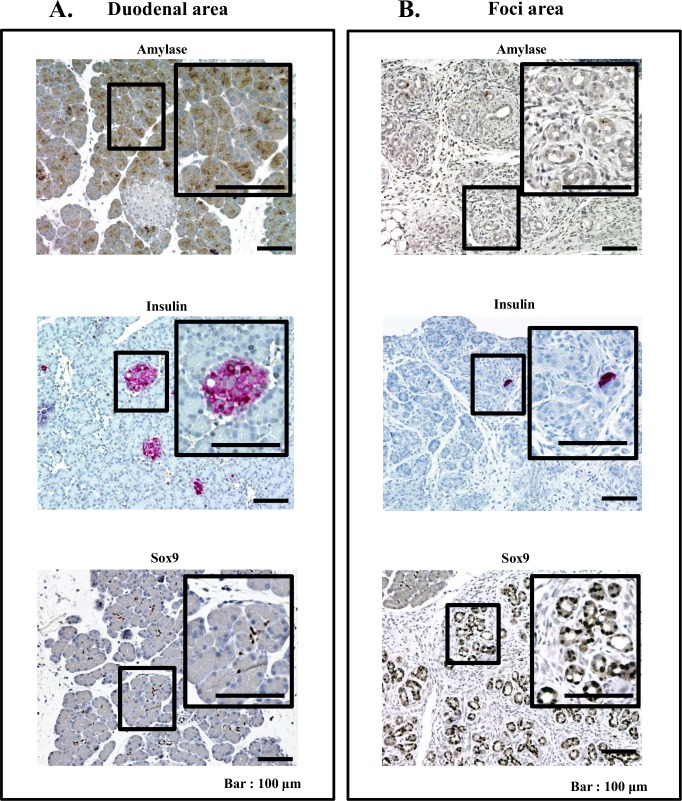
Immunohistochemical staining images of cells in duodenal and foci areas of 90%PX rats. 5 rats were sacrificed for this study. (A) Representative immune staining images of amylase (aciner cells), insulin (islet) and sox9 (centro-acinar cells) in duodenal area. (B) Representative immuno-staining images of amylase, insulin and sox in foci area. Note, unlike those in duodenal area, relatively weak staining of ductule-like cells for amylase, very little staining of islet cells for insulin and clear staining of ductule-like for sox9 cells.

To further specify the cell types in duodenal area and foci of remnant pancreas from 90% PX rat, immunohistochemical analyses of markers for PACs; amylase, for islet cells; insulin, and for progenitor cells; SOX9 were carried out (Fig 2).

In the duodenal area (Fig 2A), PACs cells were plainly stained for amylase, and centro-acinar cells were stained for SOX9 and cells in islet, which sparsely resided in this area were stained for insulin. These results suggested that duodenal area retained normal pancreas architecture.

In foci (Fig 2B), ductule-like cells were weakly stained for amylase and were mostly positive for SOX9, indicating that these cells are progenitors. Islets in this area were rarely seen.

### BrdU uptake by PACs, islet cells, and ductule-like cells after 90% PX

**Fig 3:**
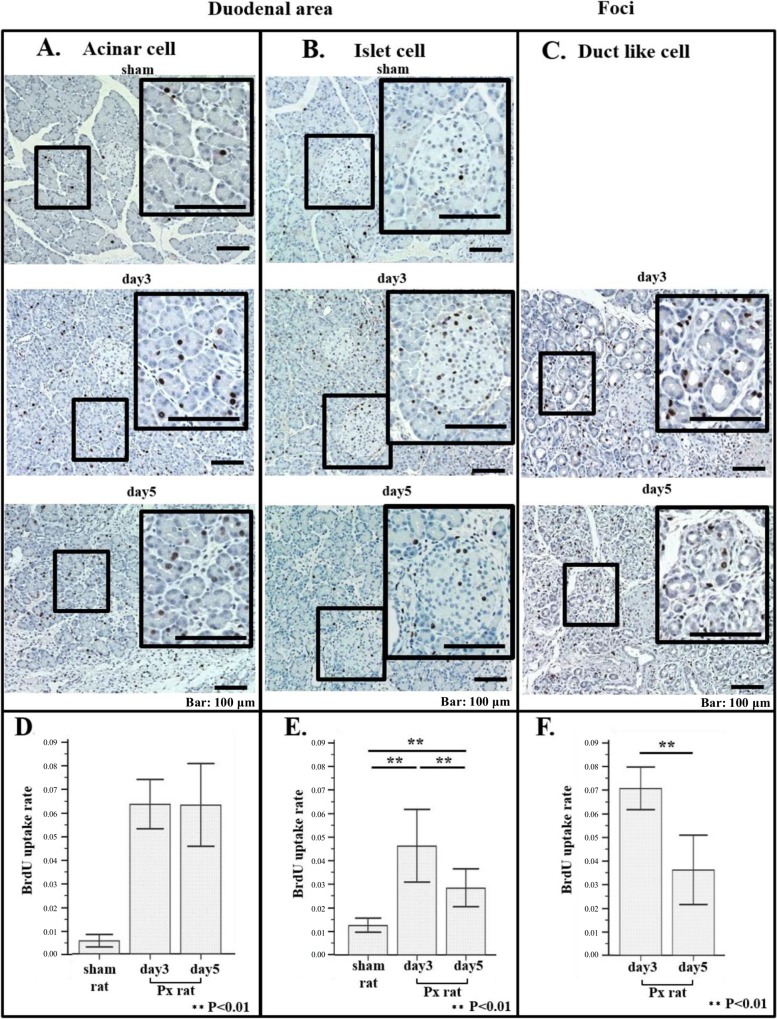
BrdU staining of acinar cells, islet cells in duodenal area and ductule-like cells in foci area of sham rat and 90% PX rat. 5 rats in each sham and 90% PX group were sacrificed in this experiment. (A) Representative immuno-staining images of acinar cells for BrdU in duodenal area of sham rat (upper panel), 90% PX rat at day3 (middle panel) and 90% PX rat at day 5 (lower panel). (B) Representative immuno-staining images of islet cells for BrdU in duodenal area of sham rat (upper panel), 90% PX rat at day3 (middle panel) and 90% PX rat at day 5 (lower panel). (C) Representative immuno-staining images of ductule-like cells for BrdU in foci area of 90% PX rat at day3 (middle panel) and at day5 (lower panel). Histogrames of average BrdU uptake rate of acinar cells (D), islet cells (E), ductule-like cells (F). BrdU uptake rates were by expressed number of positive staining per nuclei, counting 8000 nuclei per tissue specimen from each 5 rat for acinar cells, all nuclei per two specimen from each 5 rat for islet cells and 2000 nuclei per specimen from each 5 rat for ductule-like cells. Note that at day 3 and 5 after PX, BrdU uptake into acinar and islet cells significantly increased as compared to that of sham (**P<0.01) and that at day5 after PX, BrdU uptake by islet cells and ductule-like cells significantly decreased as compared that of day3 (**P<0.01).

To examine the cell proliferation in duodenal area and foci area after 90% PX, we injected BrdU iv 3h prior to harvesting pancreas from day3 rats (n = 5) and day5 rats (n = 5) tissues, and analyzed the tissue specimens for cellular uptake of BrdU by immunohistochemistry (Fig 3).

Representative staining patterns for BrdU of PACs, islet cells in the duodenal area and ductule-like cells in foci are shown in Fig 3A, 3B and 3C respectively.

In the duodenal area, numbers of BrdU positive cells of PACs and islet cells significantly increased at day3 and day5 of PX rats as compared to those of sham rats (Fig 3D), even though there is a tendency that BrdU positive islet cells decrease from day 3 to day 5 (Fig 3E).

In foci, at day 3 after PX, appreciable numbers of BrdU positive ductule-like cells was evident at day 3 and at day 5 (Fig 3C), and similarly as islet cells in duodenal area, decreased tendency of BrdU positivity was observed (Fig 3F). This indicates that the stimuli to evoke cell proliferation by surgical stress is transient.

### Increased fibrosis with proliferation of activated pancreatic stellate cells in remnant pancreas

We then postulated that stimuli such as reactive oxygen species (ROS) generated by surgical stress, induce activation of PSCs thereby some factors from aPSCs evoke the proliferation of cells in remnant pancreas. To prove this hypothesis, we first determined whether PSCs are indeed activated and proliferate in the specimen of duodenal area of rats underwent PX (day 3).

**Fig 4:**
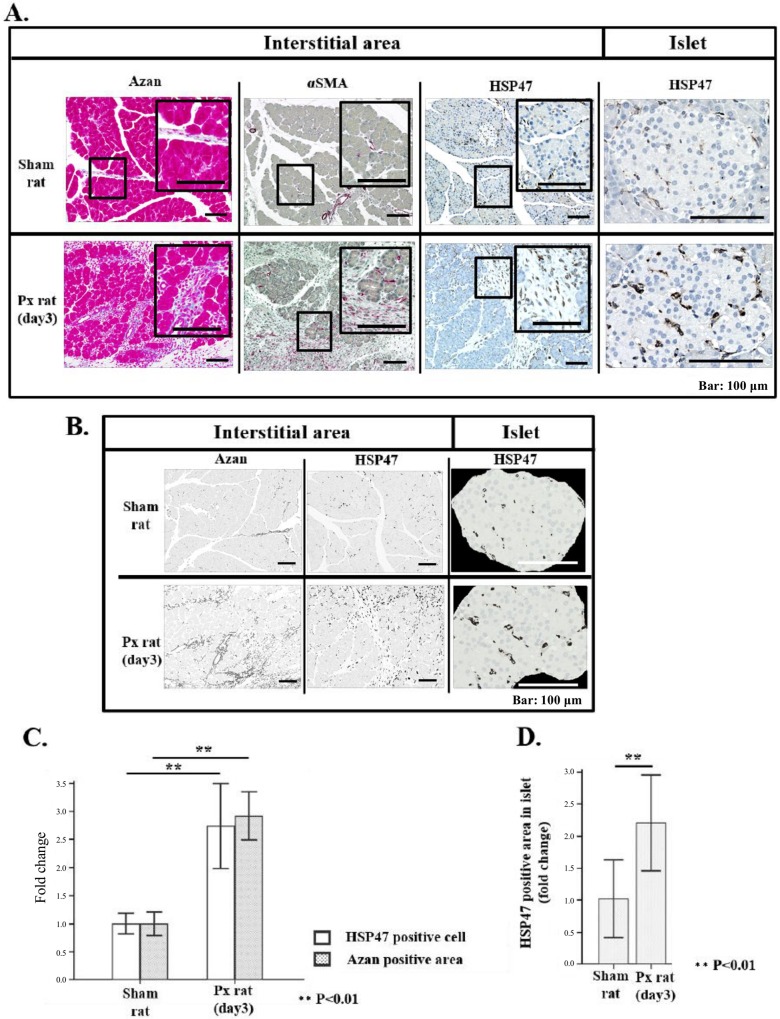
Increased extracellular matrix (ECM) deposition, αSMA positivity and HSP47 positive cells in duodenal area of remnant pancreas of 90% PX rat. On this investigation, data from 5 rats were also studied. (A) Representative Azan mallory staining images of sham pancreas and remnant pancreas at day 3 after 90% PX, and immuno-staining images of αSMA and HSP47 in interstitial area, and that of HSP47 in islet. (B) Azan positive areas and HSP47 positive cells in tissue specimen of (A),were highlighted by binarization with BzⅡ analyzer software (see for details in material & Method section.) (C)(D) Histograms of Azan positive area and HSP47 positive area in interstitial area (C), and in islet (D) employing BzⅡ analyzer software on the figures shown in Fig 4(B). Monochrome dark area in a whole specimen was computed and average value on 5 randomly selected specimen from each 5 rat was calculated. The average values of PX rats were normalized to those of sham rats. Note that both Azan positive area and HSP47 positive cell number significantly increased after PX (day3) (**P<0.01).

Representative immune staining images of duodenal area (Fig 4) clearly demonstrated increasedα-smooth muscle actin (αSMA) positive cells in the interstitial area, evidenced by Azan staining in PX rat compared to that of sham rat (Fig 4A). Though αSMA is widely used as a marker of aPSC [26,27] because vasculature wall in the tissue also showed positive staining pattern for αSMA (S1 Fig), this marker protein was considered to be unsuitable for quantitative analyses of aPSCs.

Thus, instead of αSMA, heat shock protein 47(HSP47) which is a specific chaperone for collagen secretion [23,24] was selected to identify aPSCs in the following analyses.

Immune staining of HSP47 revealed an apparent increase of HSP47 positive aPSCs in PX rat as compared to those of sham rats (Fig 4A).

HSP47 positive cells were then highlighted by image analyzing software as shown in (Fig 4B).

Quantification by brightness contrast confirmed a significantly increased HSP47 positive aPSCs after PX (Fig 4C).

Then, to determine whether fibrosis is indeed enhanced resulting from increment of aPSCs, Azan positive area was also quantitatively analyzed (Fig 4C) by image software after monochrome contrast enhancement (Fig 4B).

Then statistically significant increases of Azan positive areas after PX was confirmed (Fig 4C). Furthermore, significant correlations between HSP47 positivity and Azan stained areas was verified (data not shown).

When HSP47 positive cells per unit area of islet at duodenal site was analyzed after PX, they were also increased as compared those in sham control (Fig 4A, 4B and 4D).

**Fig 5:**
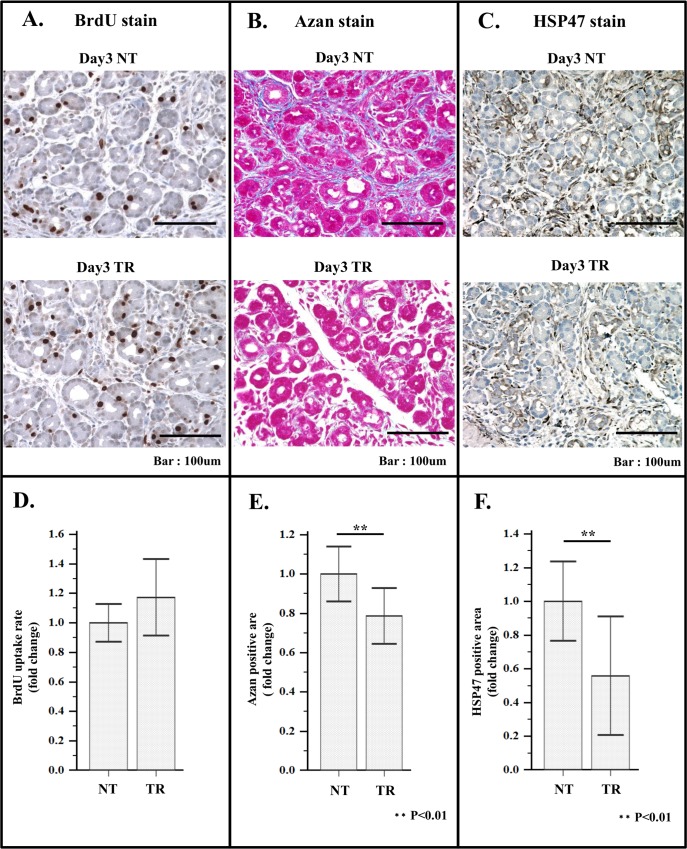
Effect of VA-lip siRNA HSP47 treatment on BrdU uptake by ductule-like cells, Azan mallory staining intensity and HSP47 expression in the foci area. (A) Representative immuno-staining images of BrdU uptake by ductule-like cells in the foci area of non-treated rat (day3 NT; upper panel), treated rat (day3 TR; lower panel). (B) Representative immuno-staining images of Azan staining in the foci area. Upper panel; non-treated rat (day3 NT), lower panel; treated rat (day3 TR). (C) Representative immuno-staining images of HSP47 stained specimen of the foci area of non-treated rat (day3 NT; upper panel), treated rat (day3 TR; lower panel). (D) Histogram of average BrdU uptake rate of non-treated rat (NT) and treated rat (TR). (E) Histogram of average Azan positive area of non-treated (NT) and treated rat (TR). (F) Histogram of average HSP47 positive area of non-treated rat (NT) and treated rat (TR). Quantification of BrdU uptake rate, Azan positive area and HSP47 positive area was performed as described above.

Regarding Azan stained fiber and the HSP47 positive cells in foci area (Fig 5B and 5C), although comparison with sham control was not practically feasible because foci area is not present in sham rat, both components were even more markedly observed than those in duodenal area of PX rat (Fig 4B and 4C upper panel).

These results suggest that the surgical procedure actually brings about activation of aPSCs in both duodenal and foci area with higher magnitude in the latter area.

### Effect of siRNA HSP47 on fibrosis formation and BrdU uptake by cells in remnant pancreas

To directly prove that the increased aPSCs in remnant pancreas are responsible for proliferation of acinar cells, islet cells and foci cells, we suppressed the activity of aPSCs by siRNA against HSP47 (siRNA HSP47) which is supposed to inhibit collagen secretion and subsequently induce apoptosis of aPSCs. Thus we first examined in vitro effects of siRNA HSP47 on aPSCs.

**Fig 6:**
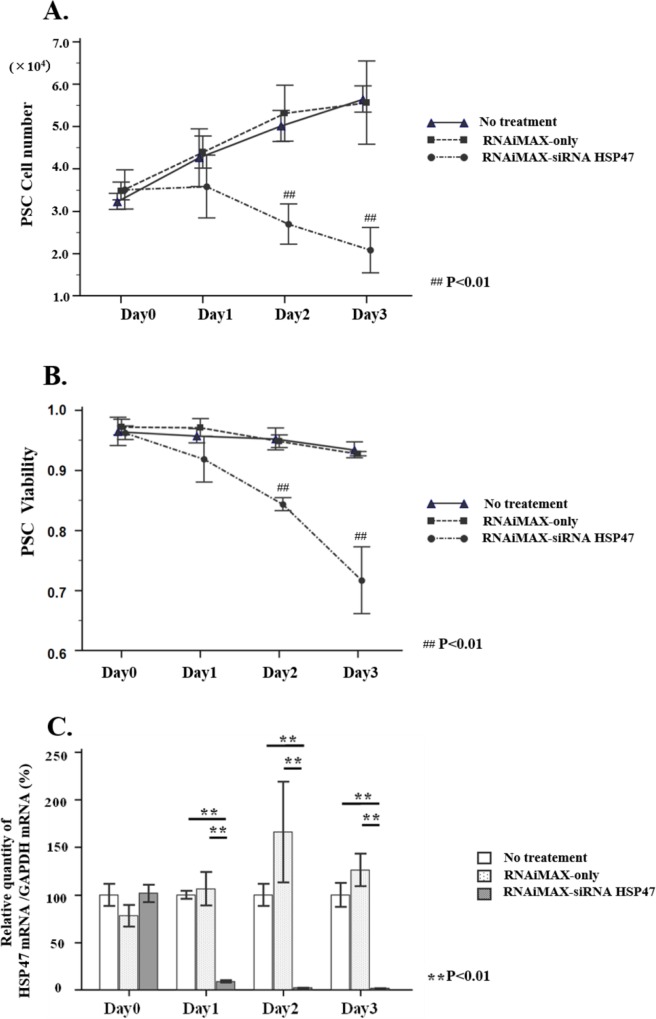
**Effect of siRNA HSP47 transfection on growth (A), viability (B) and silencing of HSP47 mRNA (C) of PSCs.** PSCs from normal rat were treated with RNAiMAX, RNAiMAX siRNA HSP47 (10nM) or medium alone and were incubated for 0–3 days at 37°C at 5×10^4^ cells density / 60mm dish for cell counting (A), for measurement of cell viability (B) by dye exclusion assay, and for quantitative measurement of HSP47 mRNA and GAPDH mRNA by qPCR method (C). Note that PSCs treated with siRNA HSP47 showed significant reduction of cell number (A) and cell viability (B) with concomitant suppression of HSP47 mRNA (C) as incubation time prolonged.

**Fig 7:**
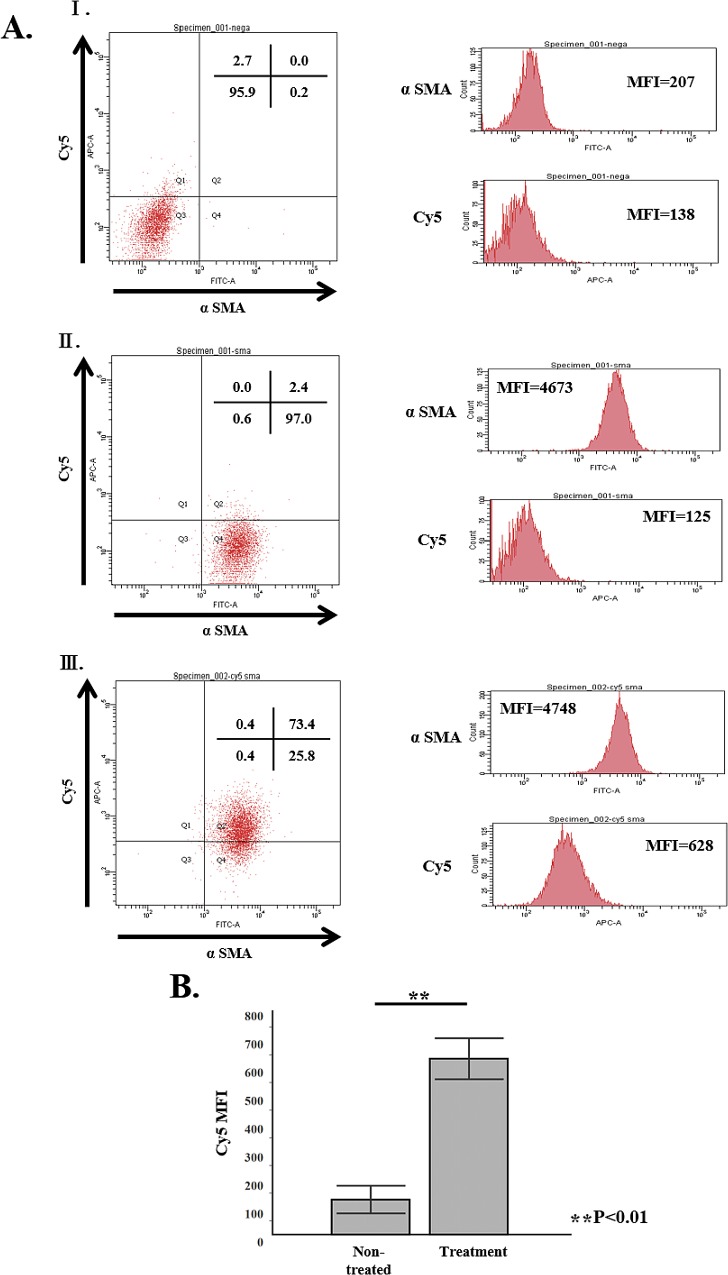
In vivo distribution of VA-lip siRNA HSP47-Cy5 toαSMA in 90% PX rat. PSC cells were isolated from remnant pancreas of three 90% PX rats according the method in previous report [24] and were subjected to FACS analysis. AⅠ; PSCs without staining (back ground control). AⅡ; PSCs from non-treated rat. AⅢ; PSCs from VA-lip siRNA HSP47-Cy5 treated rat. B. Note that almost 75% of PSCs were positive for Cy5 fluorescence with significantly higher MFI than that of non-treated rat while PSCs from non-treated rat showed essentially negative fluorescence.

Transduction of siRNA HSP47 caused time dependent suppression of HSP47mRNA and viable aPSCs (Fig 6), being consistent with our previous observation. We then performed in vivo administration of siRNAHSP47–Cy5 encapsulated in vitamin A coupled liposome (VA-lip siRNA HSP47 Cy5) to PX rat according to the protocol in S2 Fig, and confirmed successful delivery of siRNAHSP47-Cy5 to aPSCs (Fig 7).

Based on these preliminary experiments, VA-lip siRNA HSP47 was iv administered 2 times prior to and once after PX (S2 Fig) and immunohistochemical analyses of HSP47 and BrdU of acinar cells and PSCs, and Azan staining were carried out.

**Fig 8:**
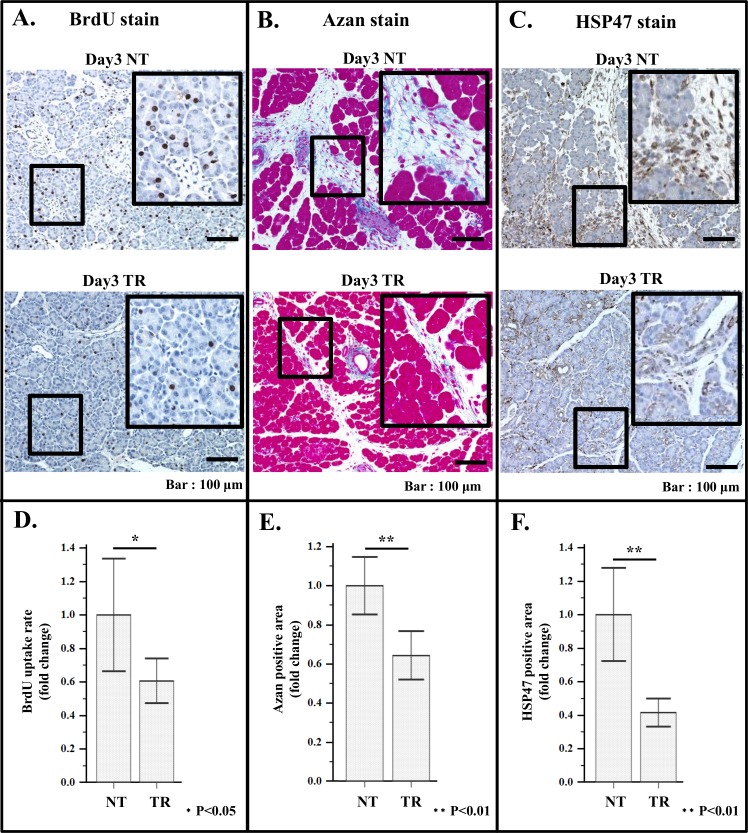
Effect of treatment with VA-liposome siRNA HSP47 on BrdU uptake, ECM deposition and HSP47 positive cell number in duodenal area. Specomen from 5 PX rats were analyzed. (A) Representative immune staining images of BrdU uptake by PACs in the remnant pancreas of non-treated rat (day3 NT; upper panel), and that of treated rat (day3 TR; lower panel). (B) Representative immuno-staining images of azan staining of the remnant pancreas. Upper panel; non-treated rat (day3 NT), lower panel; treated rat (day3 TR). (C) Representative immuno-staining images of HSP47 stained specimen of duodenal area of non-treated rat (day3 NT; upper panel), and that of treated rat (day3 TR; lower panel). (D) Histograms of average BrdU uptake rate of non-treated rat (NT) and treated rat (TR). (E) Histograms of average Azan positive areas of non-treated (NT) and treated rat (TR). (F) Histogram of average HSP47 positive areas of non-treated rat (NT) and treated rat (TR).

As expected, in the duodenal area, HSP47 stained aPSCs and Azan positive areas were significantly reduced after the treatment (Fig 8B and 8C).

Concomitantly, BrdU uptake by PACs and PSCs was also suppressed by the treatment (Fig 8A, S3 Fig), indicating that proliferation of PACs after PX is at least partly aPSCs dependent.

**Fig 9:**
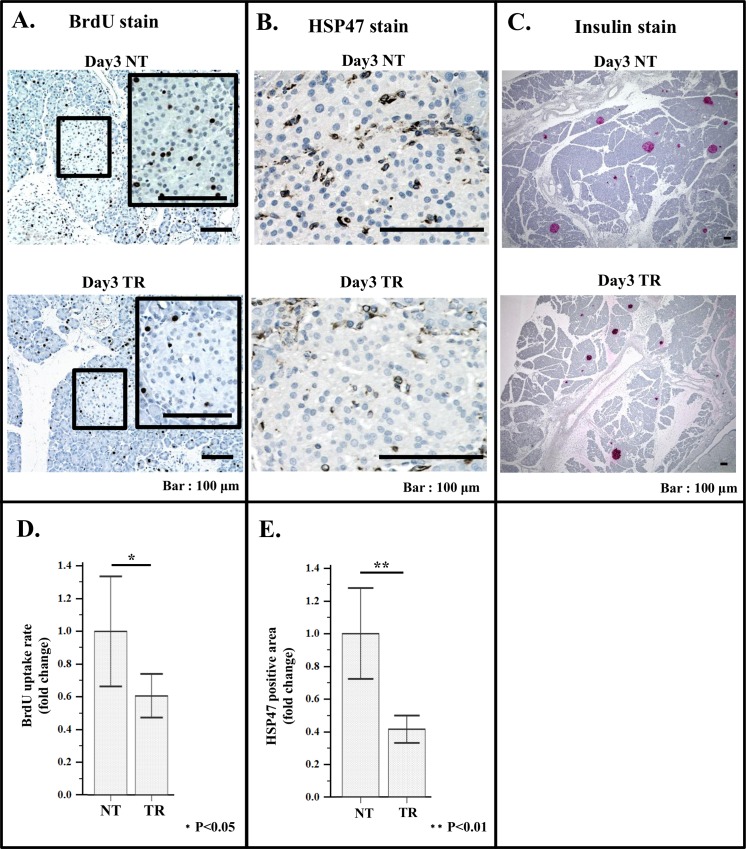
Effect of treatment with VA-lip siRNA HSP47 on BrdU uptake and HSP47 expression in islet of duodenal area. Quantification of BrdU uptake rate was carried out as described in the legend of Fig 3. Quantification of Azan positive area and HSP47 positive area was conducted as described in the legend of Fig 4. Specimens from 5 PX rats were investigated. (A) Representative immuno-staining images of BrdU positive cells in the islet of non-treated rat (day3 NT; upper panel) and that of treated rat (day3 TR; lower panel). (B) Representative immuno-staining images of HSP47 stained islet of non-treated rat (day 3 NT; upper panel) and of treated rat (day3 TR; lower Panel). (C) Representative immuno-staining images of insulin positive islet in the specimen of non-treated rat (day3 NT; upper panel) and of treated rat (day3 TR; lower panel). (D) Histogram of average BrdU uptake rate of islet cells in duodenal area of non-treated rat (NT) and treated rat (TR). BrdU uptake rate was quantified as described in the legend of Fig 3. (E) Histogram of average HSP47 positive area in islet. Quantitative analysis of HSP47 positive area was carried out according to the method described in material and method. Note that after treatment, both BrdU and HSP47 positive cells significantly decreased.

BrdU uptake by islet cells in the duodenal area, was also suppressed in parallel with the decrement of HSP positive cells in islet area by treatment with VA-lip siRNA HSP47 (Fig 9A, 9B, 9D and 9E). These results suggested that aPSCs in duodenal area are playing pivotal roles in proliferation of the cells in this area.

In foci area, however, BrdU uptake by ductule-like cells was not affected by the treatment (Fig 5A and 5D), indicating that generation of cells in foci are irrelevant to the activity of aPSCs or three times treatment with VA-lip siRNA HSP47 was not sufficient enough to reduce cell proliferation in this area.

### Emergence of areas with intermediate character between acinar and foci cells in PX rats that were, treated with VA-lip siRNA HSP47

To enhance the effect of VA lip-siRNA HSP47, an intensified protocol, 4 times treatment prior to and 2 times after PX, was employed (S2 Fig).

Histological findings in duodenal area and foci area obtained using the previous protocol was essentially reproduced by the intensified protocol, although the effects were slightly augmented with the latter protocol with marked reduction of PSCs stained for HSP47 and azan positivity in foci area (S5 Fig).

**Fig 10:**
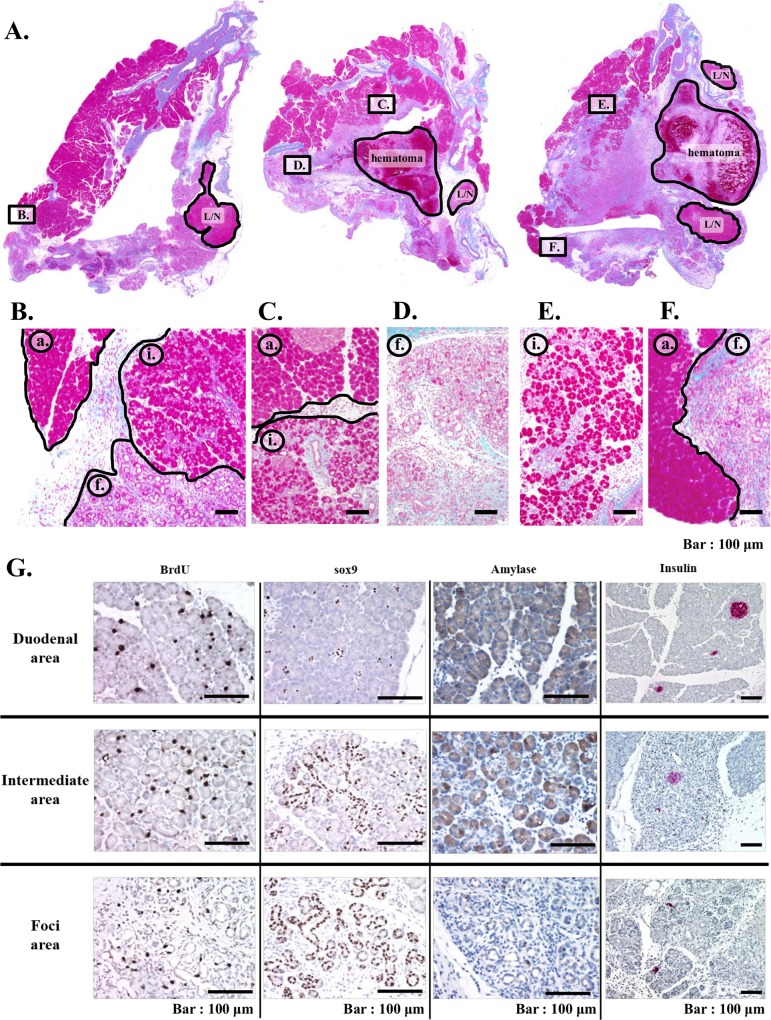
Histological characterization and immune-histochemical analyses for BrdU, SOX9, amylase and insulin expression of remnant pancreas of 90% PX rats treated with intensified protocol. In (A), gross appearances (HE staining) of remnant pancreases from three PX rats are shown. (G) Representative immuno-staining images of duodenal area, intermediate area and foci area for BrdU, SOX9, amylase and insulin. Note that intermediate area showed a feature between those of duodenal area and foci area. L/N indicates lymph node. Fig B, C, D, E and F at second line panel, intermediate area (ⓘ) were noted in all three rats in addition to acinar area (ⓐ) and foci area(ⓕ).

However, a new area with distinct histological feature from cells in duodenal area or foci area was uncovered by the intensified protocol (Fig 10ⓘ in B, C, E).

When this newly emerged area was immunohistochemically analyzed for BrdU uptake, SOX9 expression and amylase expression, the stained images were essentially the same among 3 rats and representative data are shown in Fig 10G.

Cells in this new area showed slightly higher BrdU uptake than those in the duodenal or foci area, and intermediate staining intensity for amylase between those of cells in duodenal and foci area.

SOX9 expression pattern in the new area was even more unique, exhibiting clear staining along most of the stems of the ductule with scarce positive staining of centro-acinar cells, while in the duodenal area and foci area, clear SOX9 staining of centro-acinar cells and ductule-like cells were respectively observed.

The most impressive observation was that the islets were also identified in this new area.

### Effect of aPSCs on BrdU uptake of PACs in vitro

In order to futher explore the stimulatory effect of aPSCs on proliferation of PACs, we examined BrdU uptake of PACs in the presence and absence of aPSCs by employing co-culture systems (S4 Fig).

**Fig 11:**
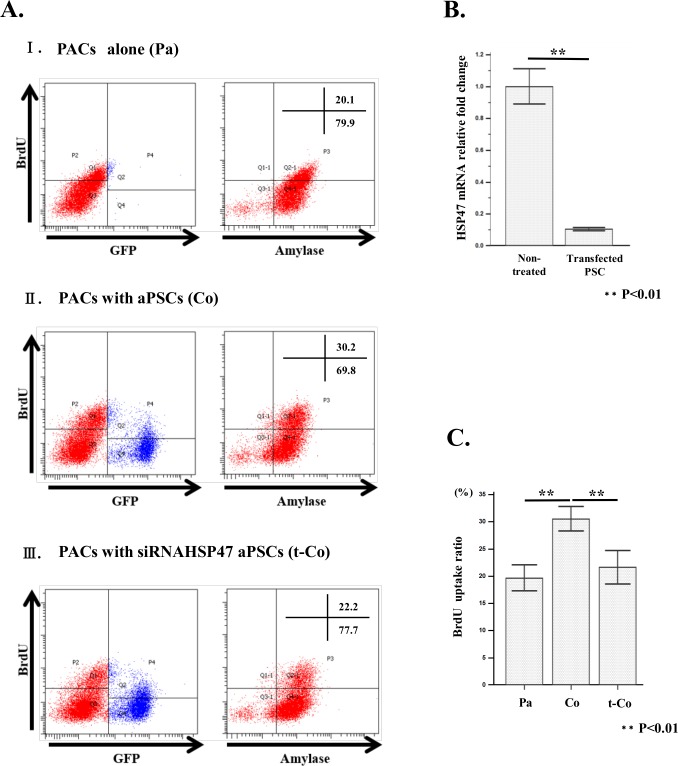
Effect of siRNA HSP47 on stimulatory activity of aPSCs to BrdU uptake by PACs in co-culture system. (A)Ⅰ BrdU uptake by PACs was analyzed by flow cytometry for GFP fluorescence, and expression of BrdU and amylase using each corresponding antibody. (A)Ⅱ BrdU uptake by PACs co-cultured with PSCs isolated from GFP rat was analyzed by flow cytometry for GFP fluorescence, and expression of BrdU and amylase using each corresponding antibody. (A)Ⅲ BrdU uptake by PACs co-cultured with siRNAHSP47 transfected PSCs from GFP rat was analyzed by flow cytometry for GFP fluorescence, and expression of BrdU and amylase using each corresponding antibody. (B) Histogram of HSP47 mRNA expression levels of siRNA HSP47 transfected and non-treated PSCs from GFP rat. Experiment was carried out three times and average value of the HSP47 mRNA levels in PSCs as measured by qPCR was calculated. Values were normalized to that of non-transfected PSCs. Note that almost complete knock down of HSP47 mRNA by siRNA HSP47. (C) Histogram of Mean fluorescence intensity (MFI) of BrdU stained PACs alone (Pa), PACs co-cultured with non-transfected PSCs (Co) and PACs co-cultured with transfected PSCs (t-Co). Experiment was carried out three times and average value of the HSP47 mRNA levels in PSCs as measured by qPCR was calculated. Note that BrdU-MFI of PACs which was significantly augmented by co-culture with PSCs was suppressed to the level of PACs alone.

BrdU uptake of PACs was significantly greater (Fig 11AⅡ and 11C) when they were co-cultured with aPSCs than with PACs alone (Fig 11AⅠ and 11C).

Introduction of siRNA HSP47 into aPSCs (Fig 11B) resulted in suppression of increased BrdU uptake under co-culture condition (Fig 11AⅢ and 11C), to the level of PACs alone, indicating that the enhancement of BrdU uptake by aPSCs is largely due to the newly released collagen from aPSCs.

## Discussion

Tissue damage caused by surgical procedure should invariably evoke acute inflammation with infiltration of inflammatory cells, which release humoral factors such as cytokines and reactive oxygen species (ROS).

Thus, the effects of these factors on the damaged tissue should be more prominent in areas adjacent to vasculature from which inflammatory cells are derived, as opposed to in the area standing apart from the vasculature.

This notion is compatible with the present finding in the remnant pancreas after partial pancreatectomy. Dramatic changes such as emergence of foci area in which SOX9 positive ductule-like progenitors [8] were proliferated was seen at the areas adjacent to communicating vessels in 90% resected specimen, whereas areas at duodenal sites located relatively apart from resection lines showed apparently normal pancreatic architecture.

In the present study, we first explored a role of aPSCs in replication of mature PACs and islet cells at duodenal sites because BrdU uptake by these cells was also augmented with concomitant increases of aPSCs after PX.

By systemic application of VA-lip siRNA HSP47 which not only resolves collagen but also induces apoptosis of aPCSs, we were able to demonstrate that aPSCs are indeed playing a stimulatory role in replication of mature PACs and islet cells in vivo since BrdU uptake of both cells was significantly suppressed by the treatment through additional role of inflammatory cells in stimulation of these cells may not be denied.

This *in vivo* observations with PACs were consistent with the results of *in vitro* experiments which demonstrated that BrdU incorporated in PACs co-cultured with aPSCs was significantly higher than that in PACs cultured alone, and was clearly suppressed when aPSCs were transfected with siRNA HSP47.

However, unlike *in vivo* experiment, in the *in vitro* co-culture experiment duration of suppression of HSP47 with siRNA was at most 36hrs and thus apoptosis of aPSCs was probably not be induced. Therefore, activity of aPSCs to promote proliferation of PACs was hypothesized to be linked to effect of collagen itself of which secretion was inhibited by siRNA HSP47.

In this regard, it may be an interesting challenge to explore the possibility in the future that MT1-MMP cleaved collagen from aPSCs stimulates replication of PACs in a paracrine manner, since our previous observation clearly demonstrated that collagen secreted from activated hepatic stellate cells (aHSCs) sustains their own survival in an autocrine manner after it is cleaved by membrane type1 metalloproteinase (MT1-MMP) [25].

Regarding the mechanism of foci area formation, there may be two possibilities; the inflammatory factors directly affect on ductal components of the remnant pancreas to become foci area, or the factors indirectly generate foci area via interventing non-ductal cells which in turn somehow affect the ductal component.

The finding in the present study seems to be compatible with the latter mechanism through the former mechanism is not completely ruled out, since the situation in the foci area where SOX9 positive ductule-like cells were surrounded by densely deposited extracellular matrix (ECM) with massive infiltration of aPSCs which should be activated by inflammatory factors. This is analogous to that of cirrhotic liver where activated hepatic stellate cells (aHSCs) stimulate proliferation of progenitor cells to form clusters within the ECM [28].

Thus, to elucidate the role of aPSCs generation of foci area we took an approach to utilize VA-lip siRNA HSP47 which not only suppresses collagen secretion from aPSCs but also induces apoptosis of aPSCs [25], thereby inhibiting total activity of aPSCs *in vivo*.

By treatment with this agent, SOX9 staining of these cells was apparently diminished in association with much less HSP47 positive cells and ECM deposition as compared to that of non-treated controls. Those data suggest a pivotal role of aPSCs in generation of foci.

Because when we treated rats with cirrhotic liver by the same modality, we clearly found differentiation of hepatic progenitor cells to hepatocytes which were liberated from niche structure consisted of hepatic stellate cells and surrounding collagen fiber (unpublished observation), we in the present study intended to yield a similar phenomenon in PX pancreas by intensified treatment.

As the results, we were able to demonstrate an emergence of new area with intermediate features between foci and duodenal area which we never identified in untreated PX pancreas. Although more extensive and direct proof is certainly needed to conclude the differentiation of pancreas progenitor cells by the treatment, present results appears to suggest that phenomenon observed in cirrhotic liver may also occur in the PX pancreas.

In conclusion, in the present investigation, we proposed a new concept of mechanism for remnant pancreas regeneration after PX with particular emphasis on the role of activated pancreatic stellate cells.

pancreatic stellate cells.

## Supporting Information

S1 Fig **Histological appearance of acini and foci area of residual pancreas, stained for Azan (A) (B), HSP47 (C) andαSMA (D) after 90% PX in rat. **Note that with anti αSMA antibody not only activated PSCs but also vascularture (D) were stained while with anti HSP47, only PSCs were specifically stained.
(TIF)

S2 Fig Treatment protocols with VA-liposome siRNA for rats undergoing 90% PX Rats were divided into three groups; I-1 first group treated with the drug once 2 days prior to PX, once just before PX, twice after PX every other day, I-2 second group treated with the drug every day 3 times prior to PX, thenafter by the same treatment schedule as I-1, third group treated with the ordinary drug twice followed by Cy5 labeled drug twice in the same schedule as I-1.
(TIF)

S3 Fig BrdU uptake by PSCs before and after the treatment in duodenal area of PX pancreas Upper panel demonstrates immunohistologically stained (red arrows) or non-stained (black arrows) PSCs in the duodenal area of pancreas before (A) and after (B) PX. Middle panel represents histogram of percentage of BrdU positive PSCs in all PSCs. Bottom panel shows actual number of PSCs counted in the specimen. Note in all panels, significant suppression of BrdU uptake by PSCs after the treatment was evident.
(TIF)

S4 Fig Procedures for co-culturing of PSCs and PACs using double chamber system Upper panel illustrates isolation procedures of PSCs from the pancreas of GFP rat and PACs from the pancreas of SD rat. Lower panel illustrates scheme of co-culturing GFP-PSCs and PACs in double chamber system.
(TIF)

S5 Fig Effect of intensified treatment on fibrosis (Azan staining) and PSCs (HSP47 staining) in foci area of 90% PX pancreas Upper panel represents typical Azan staining (left) and HSP47 staining (right) patterns of foci area of pancreas from rats treated with intensified protocol (TR) or non-treated rats (NT). Lower panel shows quantitative analysis of Azan positive and HSP47 positive areas assessed by Strata Quest Analysis software. Note that after treatment both Azan and HSP47 positive area were significantly reduced.
(TIF)
