# Supplementary figures and images for: Involvement of Pancreatic Stellate Cells in Regeneration of Remnant Pancreas after Partial Pancreatectomy
Source: PLoS One. 2016 Dec 9;11(12):e0165747. doi: 10.1371/journal.pone.0165747 (PMC5147817; doi:10.1371/journal.pone.0165747)

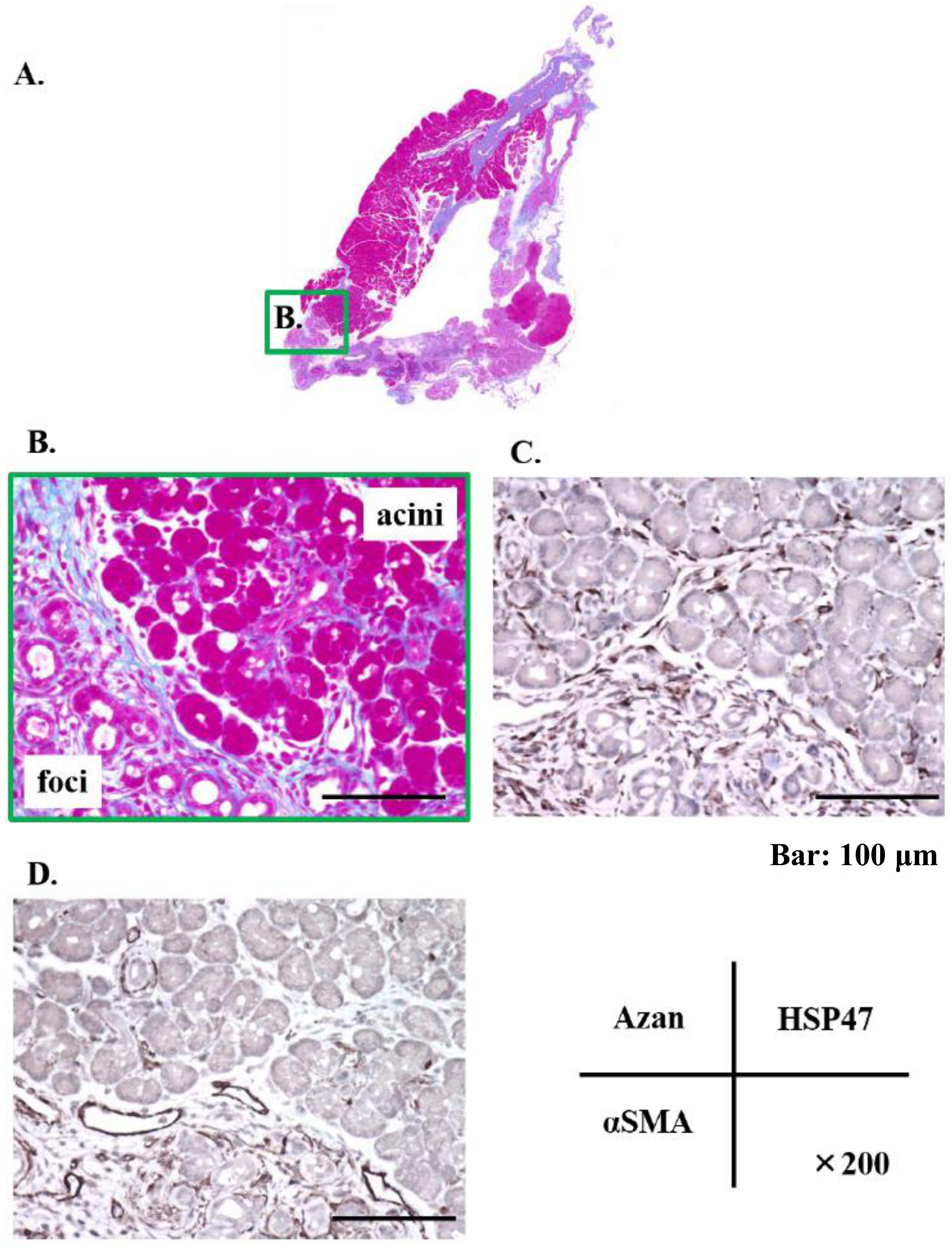

Supplement: S1 Fig — Histological appearance of acini and foci area of residual pancreas, stained for Azan (A) (B), HSP47 (C) andαSMA (D) after 90% PX in rat. Note that with anti αSMA antibody not only activated PSCs but also vascularture (D) were stained while with anti HSP47, only PSCs were specifically stained. (TIF) [file pone.0165747.s001.tif]

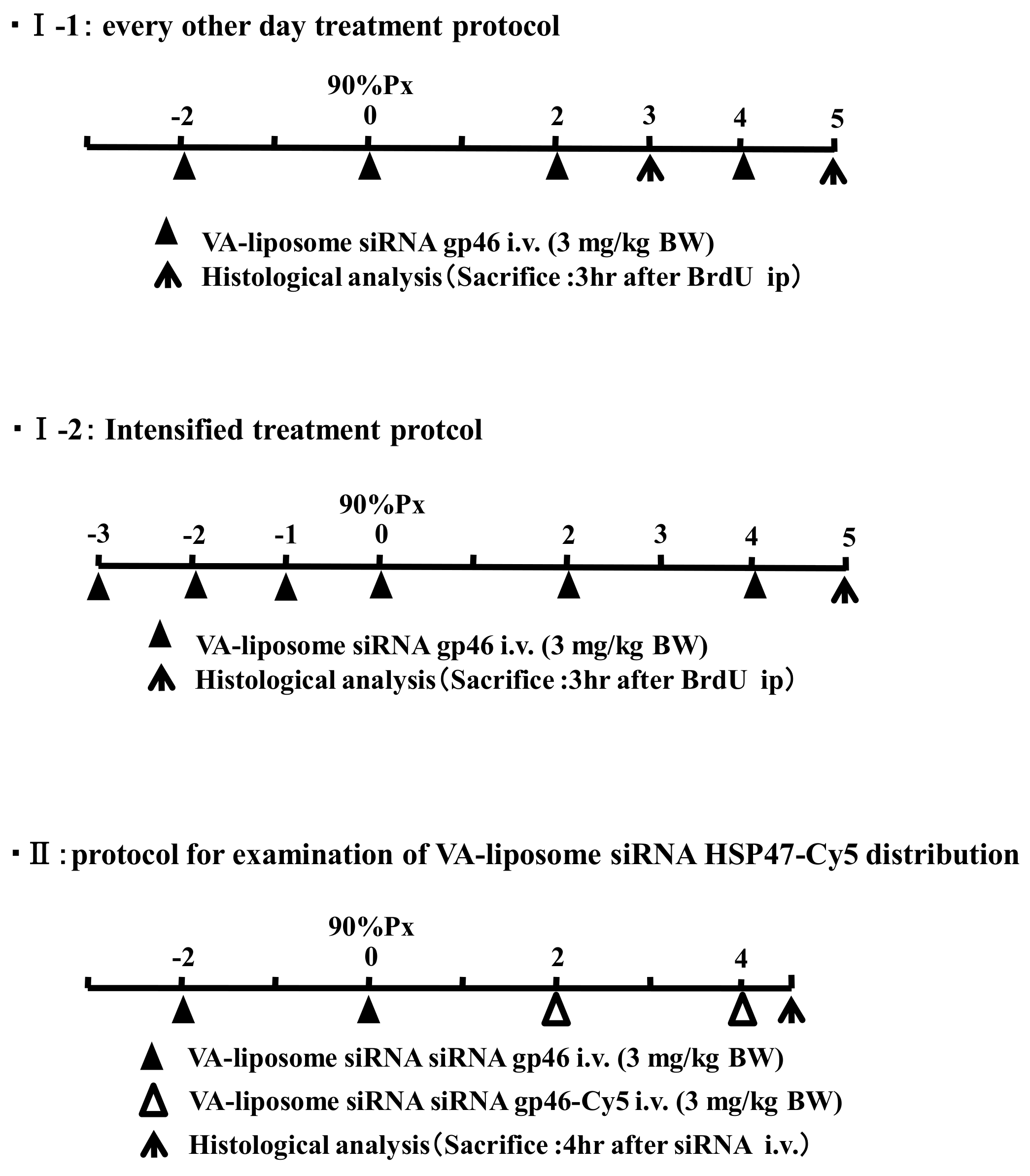

Supplement: S2 Fig — Rats were divided into three groups; I-1 first group treated with the drug once 2 days prior to PX, once just before PX, twice after PX every other day, I-2 second group treated with the drug every day 3 times prior to PX, thenafter by the same treatment schedule as I-1, third group treated with the ordinary drug twice followed by Cy5 labeled drug twice in the same schedule as I-1. (TIF) [file pone.0165747.s002.tif]

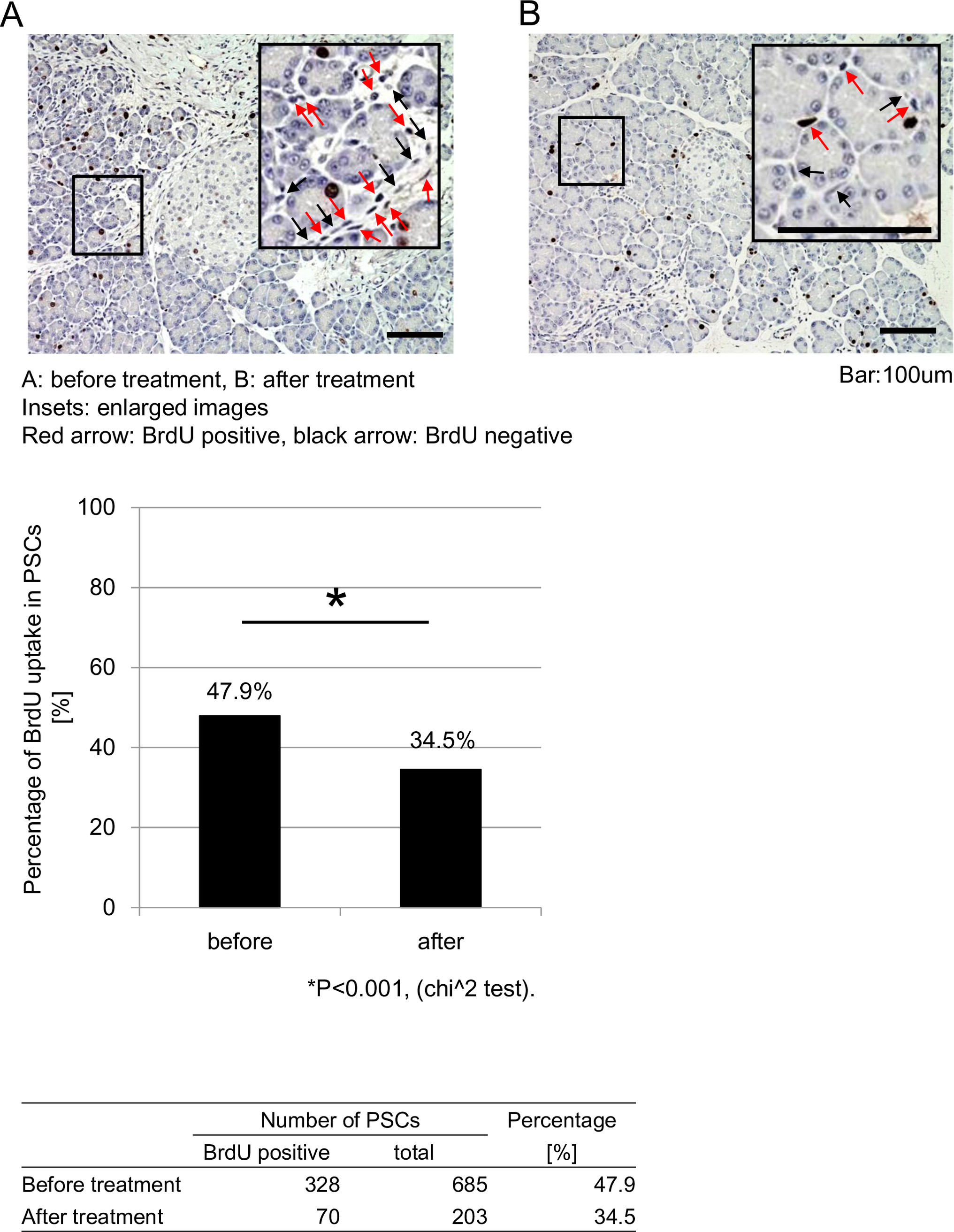

Supplement: S3 Fig — Upper panel demonstrates immunohistologically stained (red arrows) or non-stained (black arrows) PSCs in the duodenal area of pancreas before (A) and after (B) PX. Middle panel represents histogram of percentage of BrdU positive PSCs in all PSCs. Bottom panel shows actual number of PSCs counted in the specimen. Note in all panels, significant suppression of BrdU uptake by PSCs after the treatment was evident. (TIF) [file pone.0165747.s003.tif]

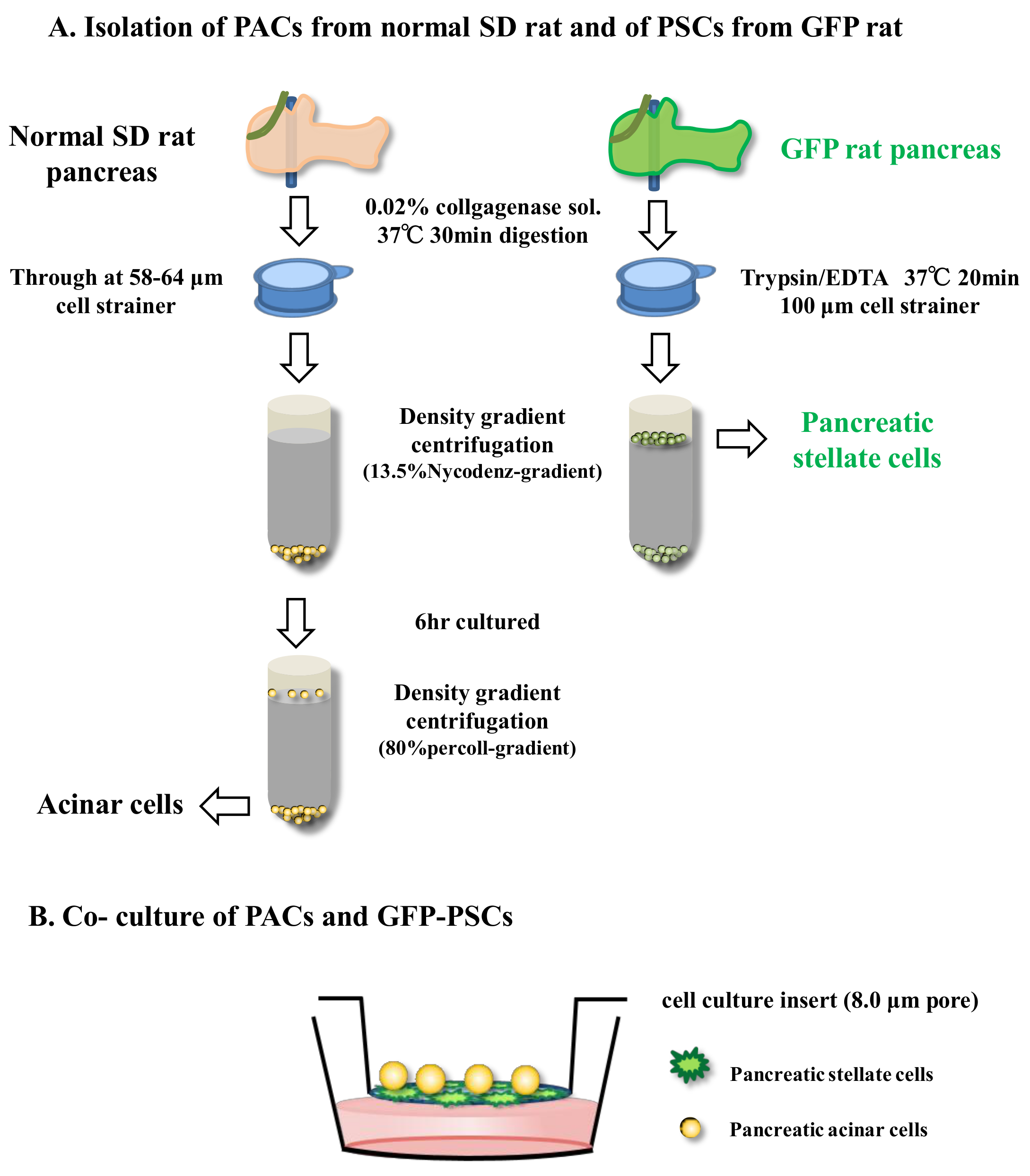

Supplement: S4 Fig — Upper panel illustrates isolation procedures of PSCs from the pancreas of GFP rat and PACs from the pancreas of SD rat. Lower panel illustrates scheme of co-culturing GFP-PSCs and PACs in double chamber system. (TIF) [file pone.0165747.s004.tif]

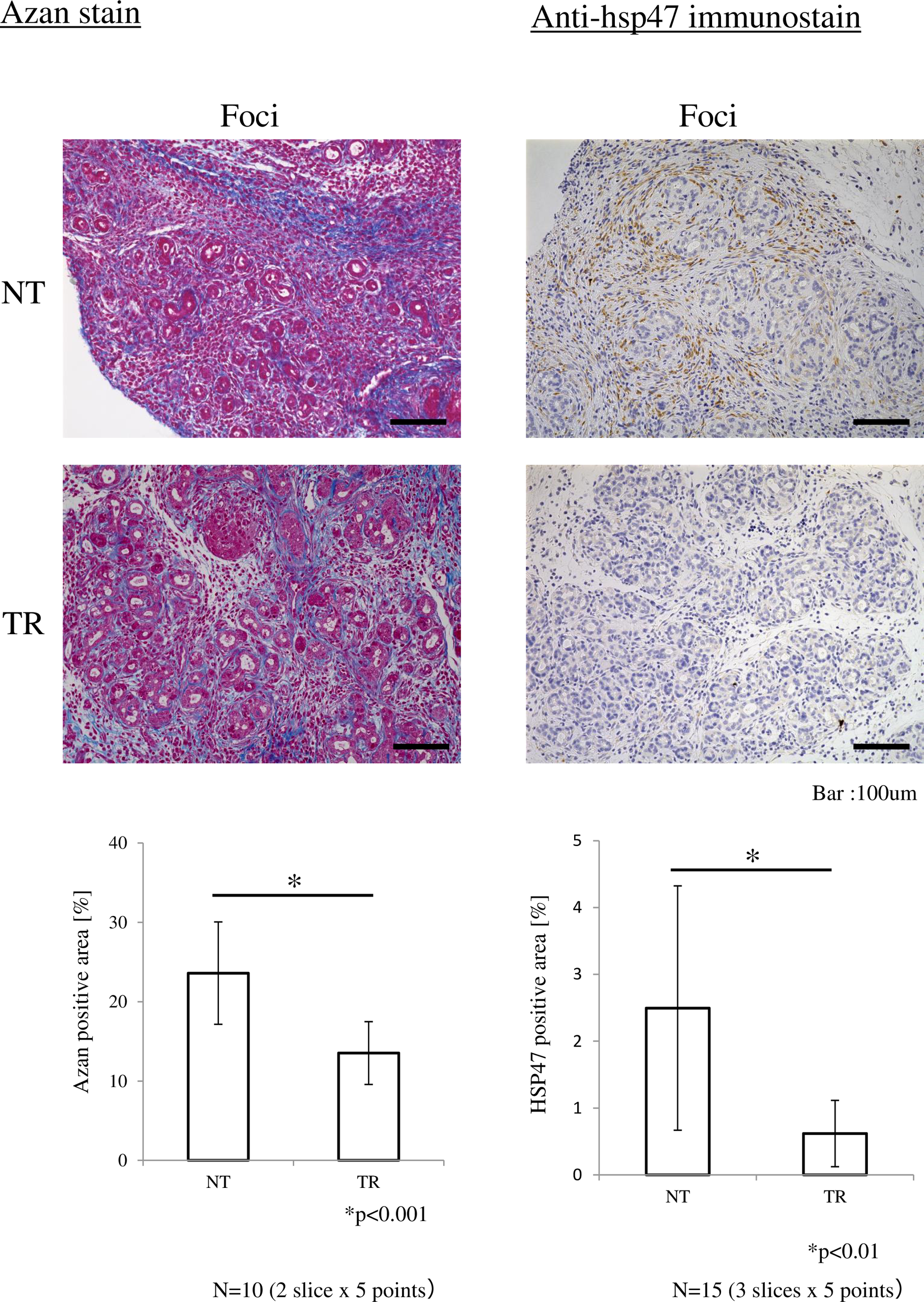

Supplement: S5 Fig — Upper panel represents typical Azan staining (left) and HSP47 staining (right) patterns of foci area of pancreas from rats treated with intensified protocol (TR) or non-treated rats (NT). Lower panel shows quantitative analysis of Azan positive and HSP47 positive areas assessed by Strata Quest Analysis software. Note that after treatment both Azan and HSP47 positive area were significantly reduced. (TIF) [file pone.0165747.s005.tif]
